# Using a Life Course Approach to Understand Nursing Assistant Perspectives on Job Satisfaction and Intent to Stay

**DOI:** 10.1093/geroni/igaf122.2139

**Published:** 2025-12-31

**Authors:** Waqar Ahmad, Jennifer Morgan, Elisabeth Burgess, Antonius Skipper, Eric Wright

**Affiliations:** A.G. Rhodes, Atlanta, Georgia, United States; Georgia State University, Atlanta, Georgia, United States; University of North Georgia, Dahlonega, Georgia, United States; Georgia State University, Atlanta, Georgia, United States; Georgia State University, Atlanta, Georgia, United States

## Abstract

Nursing home staff, particularly direct care workers, experience low job quality which includes low levels of compensation, few benefits, heavy workloads and very little career mobility. This study uses a life course framing to incorporate and amplify CNAs voices on how their working lives unfold in nursing homes (NHs). Using a modified grounded theory approach, we analyze interview data from 25 certified nursing assistants (CNAs) in two NHs as structured by previous in-depth quantitative analysis of the predictors of job satisfaction and intent to stay for nursing home staff. Our analysis provides valuable insights into a range of factors affecting CNAs’ working lives as well as the quality of care in long term care settings. We highlight how workers’ social locations are linked to job satisfaction and retention. We find evidence for the role of underexplored contingency factors, e.g., single breadwinner, health insurance, and coworker support in staying in the job and meaningful care partnership. We further find that broader life course factors and processes in the workplace, e.g., supportive leadership, equity and equality, shared culture and values, continuous education & training, and relationships in NHs significantly influence CNAs retention and interest in how care is delivered in NHs. Our findings suggest they need for key changes to organizational practice including increased access to mental health services, professional support, increased leave for caregiving and respite, improved access to high-quality health insurance and benefits that assist this workforce to deal with contingency factors and promote career mobility.

